# Dental Literature

**Published:** 1846-12

**Authors:** 


*Dental Literature.*—"A Popular Treatise on the Teeth, &c. &c., by E. G. Kelley, M. D., Member of the American Society of Dental Surgeons, and of the Massachusetts and Boston Medical Societies." A second edition of this Treatise has just been published by J. Munroe & Co , 134 Washington-st., Boston. Awhile since we noticed that dentists were considered conservators of the public health. If such is the case, this author is eminently so. He not only shows how directly ill health and imperfect physical training in childhood, operate in the production of subsequent bad teeth, but traces the connection of the latter as the chief cause of much disease and suffering, which none but physicians, and only a few of them, fully realize. It is his opinion that the great mass of dental disease may be prevented by early and proper care; that in general the teeth, if filled when decayed, as they should be, may be preserved during life. In this operation Dr. Kelley takes special pains. Many, also, who suffer from imperfect mastication without teeth, are not aware how much their health and comfort might be improved by restoring the natural complement. Dr. K. has lately established himself in Boston, in an eligible location, in Tremont Place.—*Boston Med. & Surg. Journal.*

